# Effects of propranolol in combination with radiation on apoptosis and survival of gastric cancer cells *in vitro*

**DOI:** 10.1186/1748-717X-5-98

**Published:** 2010-10-26

**Authors:** Xinhua Liao, Xiangming Che, Wei Zhao, Danjie Zhang, Houlong Long, Prakash Chaudhary, Haijun Li

**Affiliations:** 1 Department of General Surgery, First Affiliated Hospital of Medical College of Xi'an Jiao-Tong University, Yanta West Road 277, Xi'an 710061, PR China

## Abstract

**Background:**

The National Comprehensive Cancer Network (NCCN) guidelines recommend radiotherapy as a standard treatment for patients with a high risk of recurrence in gastric cancer. Because gastric cancer demonstrates limited sensitivity to radiotherapy, a radiosensitizer might therefore be useful to enhance the radiosensitivity of patients with advanced gastric carcinoma. In this study, we evaluated if propranolol, a β-adrenoceptor (β-AR) antagonist, could enhance radiosensitivity and explored its precise molecular mechanism in gastric cancer cells.

**Methods:**

Human gastric adenocarcinoma cell lines (SGC-7901 and BGC-823) were treated with or without propranolol and exposed to radiation. Cell viability and clonogenic survival assays were performed, and cell apoptosis was evaluated with flow cytometry. In addition, the expression of nuclear factor κB (NF-κB), vascular endothelial growth factor (VEGF), cyclooxygenase 2 (COX-2), and epidermal growth factor receptor (EGFR) were detected by western blot and real-time reverse transcription polymerase chain reaction (PCR).

**Results:**

Propranolol combined with radiation decreased cell viability and clonogenic survivability. Furthermore, it also induced apoptosis in both cell lines tested, as determined by Annexin V staining. In addition, treatment with propranolol decreased the level of NF-κB and, subsequently, down-regulated VEGF, COX-2, and EGFR expression.

**Conclusions:**

Taken together, these results suggested that propranolol enhanced the sensitivity of gastric cancer cells to radiation through the inhibition of β-ARs and the downstream NF-κB-VEGF/EGFR/COX-2 pathway.

## Background

Gastric cancer is estimated to account for about 10% of invasive cancers worldwide and is the second leading cause of cancer deaths. Although the incidence of gastric cancer has been decreasing, it remains a common malignancy worldwide, especially in Asia [1]. Patients with gastric cancer frequently experience recurrent tumors, even after a curative surgical resection, because gastric cancer is frequently diagnosed at an advanced stage. Surgical treatment alone is not useful for patients with local and distal recurrences. Therefore, another therapeutic modality might be useful to prevent the recurrence of advanced gastric carcinoma. The National Comprehensive Cancer Network (NCCN) guidelines on gastric cancer treatment recommend radiotherapy as a standard treatment for patients with a high risk of recurrence, which is also supported by the clinical trial INT0116 [2]. Because gastric cancer has limited sensitivity to radiotherapy, a radiosensitizer is needed to overcome this problem.

It has been reported that antagonists of cyclooxygenase 2 (COX-2), epidermal growth factor receptor (EGFR), and vascular endothelial growth factor (VEGF) can act as radiosensitizers to enhance therapeutic sensitivity in many tumors [3-6]. Although associated with cell proliferation, invasion, angiogenesis and metastasis, nuclear factor κB (NF-κB) has been closely linked with radioresistance in multiple tumors [7,8]. Numerous studies suggest that prosurvival signaling mediated by NF-κB is linked to radiation resistance and poorer clinical outcomes among many cancers. Helen *et al*. reported that activation of β-adrenoceptors (β-ARs) and the subsequent stimulation of COX-2 and VEGF expression was perhaps an important mechanism in the tumorigenic action of nicotine in colon tumor growth [9]. It is not yet known whether propranolol (a β-AR antagonist) can be used as a radiosensitizer. The goal of this study was to investigate radiosensitizing activities of propranolol in human gastric cancer cell lines and to determine its precise signaling pathway.

## Methods

### Cell culture and drug treatment

Two human gastric adenocarcinoma (HGC) cell lines, BGC-823 and SGC-7901, were established in the People's Hospital of Peking University and China and No.6 Hospital of Shanghai, China, respectively. These two human gastric cancer cell lines were obtained from the Medical Center Laboratory of Xi'an Jiaotong University (Xi'an, China). Both cell lines were cultured in complete Dulbecco's modified eagle medium (Gibco, Grand Island, NY) containing 10% (v/v) heat-inactivated fetal bovine serum (Gibco, Grand Island, NY), penicillin (100 U/mL) and streptomycin (100 mg/mL), and they were maintained in a 37°C humidified incubator supplying 5% CO_2_. When cells reached the logarithmic phase, they were treated with isoproterenol (25 μmol/L) or propranolol (50 μmol/L). The concentrations of drugs were chosen from our previous research. The β-AR antagonist propranolol and the β-AR stimulator isoproterenol were purchased from Sigma Chemical. After 24 h of drug exposure, untreated and drug-treated cultures were irradiated at different doses (0, 2, 4, 6, 8 and 10 Gy). X-irradiation was performed with an X-ray generator (Elekta Precise Linear Accelerator, UK) at 4 Mev with a source-skin distance of 100 cm and at a dose rate of 200 cGy/min.

### Cell survival analysis

Colony formation assays were used to quantify the cytotoxicity of gastric cancer cells induced by treatments. The cells were plated in six-well plates (Costar, USA) at low densities. After overnight culture, the cells were treated as described above. The treated cells were cultured until colonies formed. The colonies were washed with PBS and stained with a crystal violet dye. The surviving fraction of each irradiation dose was calculated as the total number of colonies/(total cells inoculated×plating efficiency). A dose-survival curve was obtained for each experiment and used for calculating several survival parameters. Parallel samples were set at each radiation dosage.

### Cell apoptosis analysis

To detect phosphatidylserine externalization (on the surface of cell membrane), an indicator of early apoptosis, flow cytometry (FCM, BD Biosciences, USA) was performed with PI and fluorescein isothiocyanate (FITC)-labeled Annexin V (Joincare Biosciences, Zhuhai, China) [10]. After treatment, the remaining intact cells were incubated at 37°C for 24 hr, and then the cells were washed with cold PBS at 4°C. After centrifugation at 1500 rpm for 5 min, 500 μL of 1×binding buffer, 5 μL of FITC-labeled Annexin V and 10 μL of PI were added to the cell suspension and gently mixed. After incubation at 25°C for 10 min in the dark, the cells were analyzed by FCM.

### Real-time reverse transcription polymerase chain reaction (real-time RT-PCR)

Total RNA was extracted from cultured cells by Tri-Reagent (Sigma, MO, USA). To eliminate DNA contamination, extracted RNA was treated with a genomic DNA elimination mixture. Subsequently, the purified RNA was reverse transcribed to cDNA. Expression of β1- and β2-AR mRNA was quantified by RT-PCR (Applied Biosystems, Inc., Foster City, CA). The expression of COX-2, VEGF and EGFR was quantified using a real-time RT-PCR kit from Takara (Takara Biochemicals, Japan). Briefly, following a pre-heating step at 95°C for 10 min, the reaction was carried out using an Icycler (Bio-Rad, Hercules, CA) at a melting temperature of 95°C for 15 sec and an annealing temperature for 1 min for 40 cycles. The primer sequences and annealing temperatures for the six genes studied are given in Table 1. Primers were designed according to Genbank, NCBI. For validation, each experiment was done in triplicate.

**Table 1:** The primer sequences and annealing temperatures for the seven genes studied

Gene	Annealing temperature(°C)	Primer sequence		Amplicon (bp)	**Accession No**.
β-actin	60	Forward Reverse	ATCGTGCGTGACATTAAGGAGAAG AGGAAGGAAGGCTGGAAGAGTG	179	NM_001101
β_1_-AR	60	Forward Reverse	GGGAGAAGCATTAGGAGGG CAAGGAAAGCAAGGTGGG	270	NM_000684
β_2_-AR	60	Forward Reverse	CAGCAAAGGGACGAGGTG AAGTAATGGCAAAGTAGCG	334	NM_000024
COX-2	57	Forward Reverse	TTGACCAGAGCAGGCAGATG CCAGAAGGGCAGGATACAGC	171	NM_000963.2
VEGF-A	57	Forward Reverse	CTGGGCTGTTCTCGCTTCG CTCTCCTCTTCCTTCTCTTCTTCC	140	NM_001025370.1
EGFR	53	Forward Reverse	AGG ACA GCA TAG ACG ACA C AGG ATT CTG CAC AGA GCC A	90	NM_005228.3

### Western blot assay

The primary antibodies recognizing the β1-adrenergic receptor and β2-adrenergic receptor were purchased from Abcam (Cambridge, Mass). Antibodies recognizing COX-2, VEGF, NF-κB (p65), and EGFR were purchased from Santa Cruz Biotechnology (Santa Cruz, CA). The nitrocellulose membrane was purchased from Millipore (Bedford, Mass). The BCA assay kit and the chemiluminescence kit were purchased from Pierce (Rockford, Ill). Equal amounts of protein (20 mg) of each sample, quantified by the Bradford method, were electrophoresed on 10% SDS-PAGE and electrotransferred onto nitrocellulose membranes (400 mA for 2 hr) using a Bio-Rad Mini PROTEAN 3 System (Hercules, CA) according to the standard protocol. Wet transfer was used for EGFR protein, and semi-dry transfer was used for other proteins. The nitrocellulose membranes were then blocked with TBS containing 10% milk powder and 0.1% Tween-20 at 37°C for 4 hr. Subsequently, the membranes were incubated with a 1:200 dilution of the primary antibodies for β1-AR, β2-AR, COX-2, VEGF, EGFR and NF-κB (p65), and a 1:500 dilution of anti-β-actin at 4°C overnight. An antibody against rabbit or mouse IgG was used as the secondary antibody corresponding to the appropriate primary antibody. Immunopositive bands were examined by an enhanced chemiluminescence (ECL) detection system (Amersham Bioscience, Piscataway, NJ, USA), and the images were transferred onto an X-ray film according to the manufacturer's instructions.

### Statistical analysis

The results were expressed as the mean ± S.D. Statistical differences were estimated by one-way analysis of variance (ANOVA) followed by Dunnett's test. Those p values that were less than 0.05 were considered statistically significant. Analysis of the data and plotting of the figures were performed with the aid of software (Origin Version 7.5 and SPSS Version 13.0).

## Results

### Expression of β1- and β2-adrenergic receptors in SGC-7901 and BGC-823 cells

**Figure 1:**
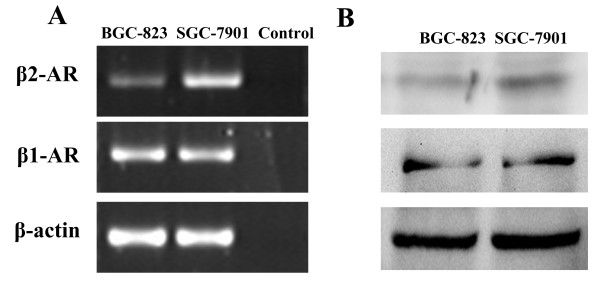
**Expression of β-ARs in human gastric cell lines SGC-7901 and BGC-823 by RT-PCR and western blotting**. (A) Expression of β-ARs in human gastric cell lines SGC-7901 and BGC-823 at the mRNA level by RT-PCR. Both of cell lines expressed β1- and β2-AR mRNA (contol group had no cDNA). (B) Expression of β-ARs in human gastric cell lines SGC-7901 and BGC-823 at the protein level by western blotting. Both of cell lines expressed the proteins of the β1- and β2-ARs.

Because propranolol is a β-adrenergic receptor antagonist, the expression of β1- and β2-ARs was determined at both the mRNA and protein level in SGC-7901 and BGC-823 cells by RT-PCR and western blot. Our results showed that β1- and β2-adrenergic receptors could be detected at both the mRNA and protein level in both cell lines. Figure 1 shows that expression of β1- and β2-adrenergic receptors in SGC-7901 cells was higher than that in BGC-823 cells.

### Dose-survival curves of gastric cancer cells after different doses of irradiation with or without propranolol pre-treatment

**Figure 2:**
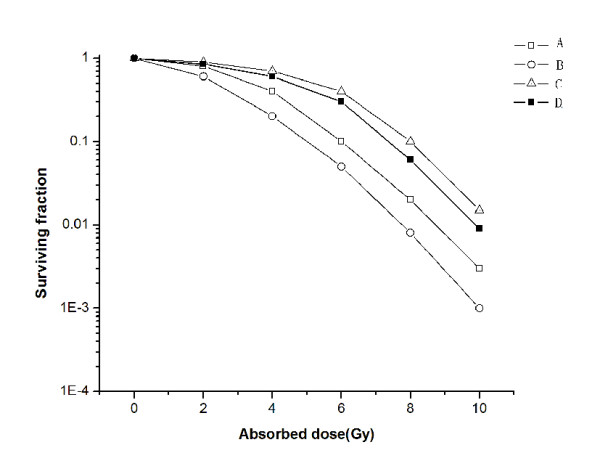
**Dose-survival curves of BGC-823 and SGC-7901 cells after different doses of radiation with or without propranolol (50 μmol/L) 24 hr before irradiation**. Propranolol administration before irradiation of BGC-823 (A) and SGC-7901 (B) cells; BGC-823 (C) and SGC-7901 (D) cells with irradiation. Compared with the irradiation-only groups, the cells exposed to propranolol before irradiation were more sensitive to irradiation.

To analyze the survival capability of gastric cancer cells against propranolol induced cell death, the cell lines SGC-7901 and BGC-823 were treated with propranolol (50 μmol/L) 24 hr prior to irradiation, and the surviving fraction of cells was determined in a clonogenic survival assay. The survival curve of control and propranolol-treated SGC-7901 and BGC-823 cells after irradiation is shown in Figure 2. A significant difference in the colony forming rate was found in combination with irradiation and propranolol at 50 μmol/L in SGC-7901 and BGC-823 cells (p < 0.01) compared with irradiation alone. Pre-treatment of SGC-7901 and BGC-823 cells with 50 μmol/L propranolol prior to irradiation resulted in a significant decrease in the surviving fraction of cells and an increase in radiation sensitivity at low irradiation doses. The decreased survival rate in propranolol-treated cells indicated that treatment with propranolol significantly improved the biological effect of irradiation.

### Propranolol enhances X-ray-induced gastric cancer cell death by promoting apoptosis

**Figure 3:**
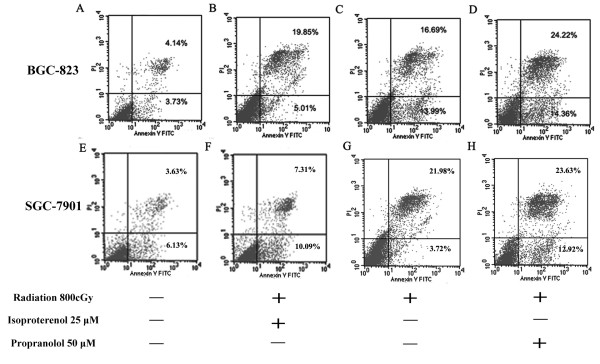
**Apoptosis induction by isoproterenol or propranolol in combination with irradiation in BGC-823 and SGC-7901 cells**. There was an increasing rate of apoptosis in gastric cancer cell lines in response to the following treatments: isoproterenol before irradiation, irradiation only, and propranolol before irradiation. The two cell lines that were treated with propranolol before irradiation had the highest apoptosis rates.

To determine whether the radiosensitizing effect of propranolol is mediated by apoptosis, the effect of propranolol on the induction of apoptosis was examined using flow cytometric (FCM) analysis with Annexin V-PI staining. After propranolol pre-treatment (50 μM for 24 hr) and following irradiation, FCM demonstrated an increase in Annexin-V positive apoptotic BGC-823 and SGC-7901 cells compared with irradiation alone. Figure 3 shows that when cells were subjected to 800 cGy irradiation in addition to propranolol, compared with irradiation alone, the apoptosis rates were 39.73 ± 2.23% vs. 25.20 ± 0.99%, p < 0.01 (SGC-7901) and 38.69 ± 1.87% vs. 31.10 ± 1.83%, p < 0.01 (BGC-823). These data suggest that propranolol can significantly increase cell death in both cell lines.

### The effects of propranolol on radiation-induced gene expression in gastric cancer cells

**Figure 4:**
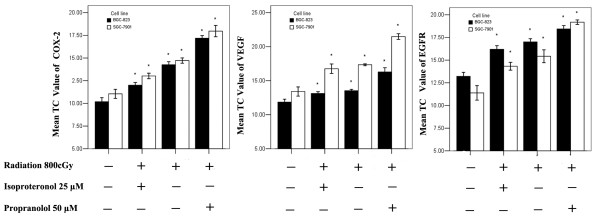
**Quantification of mRNA expression of different genes**. Analysis of mRNA expression of COX-2, VEGF and EGFR was performed on four groups: control, radiotherapy (800 cGy) after isoproteronol (25 μM), radiotherapy (800 cGy) and radiotherapy (800 cGy) after propranolol (50 μM) using an iCycler (Bio-Rad). Expression of COX-2, VEGF and EGFR was reduced significantly in different groups (*p < 0.05 versus the control group).

**Figure 5:**
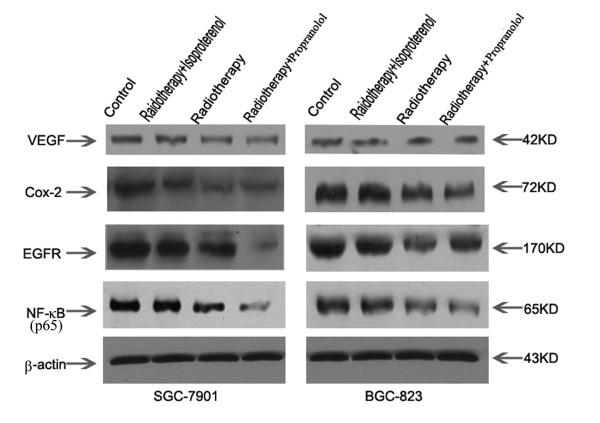
**Effects of isoproterenol, propranolol and/or radiotherapy on COX-2, VEGF, EGFR and NF-κB (p65) proteins**. SGC-7901 (A) and BGC-823 (B) cells were treated with/without isoproterenol or propranolol for 24 hr prior to radiotherapy (800 cGy). The protein levels of COX-2, VEGF, EGFR and NF-κB were analyzed by western blot.

As measured by real-time RT-PCR and western blot assay, we found that irradiation (last three groups) of BGC-823 and SGC-7901 cells down regulated the levels of NF-κB (p65) at the protein level with a subsequent decrease in COX-2, VEGF and EGFR mRNA levels (Figure 4) and proteins (Figure 5) compared with controls. After pre-treatment with propranolol, the expression of NF-κB, COX-2, VEGF, and EGFR was decreased and significantly lower than the irradiation-only group. In addition, the pre-treatment of isoproterenol had the opposite effect and reduced the downregulation of gene expression caused by irradiation. These results clearly suggested that treatment with propranolol significantly improved the biological effect of irradiation and down regulated expression of the COX-2, VEGF and EGFR genes in gastric cancer cells, which was mainly due to the decrease in expression of NF-κB via inhibited β-ARs.

## Discussion

Gastric cancer is one of the major causes of cancer mortalities in the world, and radiotherapy is an important treatment for gastric cancer patients with a high risk of recurrence. As we know, radiosensitizers have played a key role in radiotherapy. In recent years, many researchers have focused on antagonists of VEGF, COX-2 and EGFR expression as radiosensitizers [3-6], all of which have the ability to enhance the sensitivity to radiation. Helen *et al*. reported that β-ARs and the downstream COX-2 and VEGF genes played an important role in colon tumor growth [9]. This suggests that propranolol (β-AR antagonist) may act as a radiosensitizer of gastric cancer. To our knowledge, this study was the first to determine the propranolol radiosensitizing activities in human gastric cancer cell lines and to investigate its precise signaling pathway.

Based on results from the colony-forming assays, propranolol and irradiation cooperated to yield fewer and smaller colonies, suggesting that there was radiosensitization in the SGC-7901 and BGC-823 cell lines. In addition, propranolol showed a synergism of growth inhibition in combination with irradiation in SGC-7901 and BGC-823 cells. On the contrary, isoproterenol demonstrates anti-irradiation effects, which led to higher survival rates than treatment with irradiation only by using isoproterenol before irradiation. Furthermore, the apoptosis assays show that the combination of propranolol and irradiation leads to higher apoptosis rates compared with irradiation only. In addition to this, less apoptosis was observed in comparison to the irradiation-only group caused by pre-treatment of isoproterenol. The apoptosis rates of these three groups are higher than the control group. These results suggest that propranolol (β-adrenergic receptor antagonist) might be a useful irradiation sensitizer in gastric cancer therapy. Guidelines of the NCCN on gastric cancer treatment show that radiotherapy is a standard treatment for gastric cancer patients. Taken together, radiotherapy in combination with propranolol can be more useful for patients with a high risk of recurrence in gastric cancer.

Investigation of the specific mechanisms of NF-κB activation by radiation is currently a rapidly expanding field of research. It has been reported that NF-κB plays a key role in cellular protection against a variety of genotoxic agents including irradiation [11]. Radiation activates NF-κB activity in cancer cells, thus making the cells radioresistant [12]. Activation of NF-κB by various stimuli, including inflammation, stress and radiation, involves degradation of the inhibitory subunit and translocation of activated NF-κB to the nucleus to regulate transcription [13,14]. Our results demonstrated that treatment of BGC-823 and SGC-7901 cells with propranolol reduced the levels of NF-κB, suggesting that cellular radiosensitivity is increased by propranolol-induced NF-κB inhibition. It has been shown that NF-κB is involved in the modulation of expression of several proinflammatory, prometastatic and proangiogenic genes, including COX-2, EGFR and VEGF [15]. Anti-apoptotic COX-2 is an enzyme that converts arachidonic acid to prostaglandins and is inducible by radiation [16,17]. It is reported that COX-2 inhibitors act as radiosensitizers in brain tumors [3]. EGFR is a member of the ErbB family of receptors. Its stimulation by endogenous ligands, EGF or transforming growth factor-alpha (TGF-α), results in activation of intracellular tyrosine kinases and promotes cell cycle progression. EGFR was shown to play an influential role not only in cellular growth and differentiation in healthy tissues, but also in tumorigenesis and the progression of malignant disease [18]. Now, in most studies, EGFR inhibitors are given as a radiosensitizer to enhance the effect of radiotherapy [19-21]. VEGF is thought to be a critical angiogenic factor for endothelial cell proliferation and blood vessel formation. Thus, interfering with VEGF signaling has become a major strategy to inhibit tumor growth and spread [22,23]. It has been shown that anti-angiogenic agents combined with radiotherapy improved tumor oxygenation and increased treatment efficacy by killing both cancer and endothelial cells [24]. It is well accepted that the expression of EGFR, VEGF, and COX-2 is regulated by NF-κB [25-27]. In the present study, propranolol radiosensitization effects were found to be associated with changes in the levels of COX-2 and EGFR and VEGF signaling molecules. It was observed that propranolol can act as a radiosensitizer, which occurred via inhibition of β-ARs and subsequent reduced NF-κB DNA-binding activity, which concomitantly inhibited the expression of COX-2, EGFR and VEGF genes. In this way, propranolol can enhance the effect of radiotherapy on gastric cancer.

These findings, along with the present experimental data, strongly suggest that propranolol, a β-adrenergic receptor antagonist, plays an important role in the radiotherapy of gastric cancer. The present study demonstrates for the first time that β-adrenergic inhibition can enhance the effect of radiotherapy on gastric cancer cells *in vitro *through the downregulation of NF-κB and modulation of downstream COX-2, EGFR and VEGF gene expression. Furthermore, there is an opposite effect caused by isoproterenol (β-adrenergic receptor agonist) administration. These data suggest that blockade of β-AR-stimulated signaling pathways could have therapeutic implications for augmenting the sensitivity of radiotherapy on gastric cancer.

## Conclusion

In conclusion, the addition of propranolol to radiotherapy led to a decrease in gastric cancer cell survival *in vitro*. Adding the drug will enhance the sensitivity of gastric cancer cells to radiation through the inhibition of β-ARs and the downstream NF-κB -VEGF/EGFR/COX-2 pathway.

## Competing interests

The authors declare that they have no competing interests.

## Authors' contributions

XC and XL designed the study, coordinated the work and drafted the manuscript. HLo, HLi and PC did the cytological work, helped with irradiation tests, performed Western blots and PCR. WZ coordinated the work, interpreted the data and helped drafting the manuscript. All authors read and approved the final manuscript.

## References

[B1] David M.R The epidemiology of gastric cancer Gastric Cancer 2002 5 5 11 10.1007/s10120-002-0203-6 12772880

[B2] Macdonald JS Smalley SR Benedeth J Estes N Haller DG Ajani JA Gunderson LL Jessup M Martenson JA Postoperative combined radiation and chemotherapy improves disease-free survival (DFS) and overall survival (OS) in resected adenocarcinoma of the stomach and gastroesophageal junction: Update of the results of Intergroup Study INT-0116 (SWOG 9008) Gastrointestinal Cancers Symposium 2004 Abstract 6

[B3] Sminia P Kuipers G Geldof A Lafleur V Slotman B COX-2 inhibitors act as a radiosensitizer in tumor treatment Biomed Pharmacother 2005 59 272 275 10.1016/S0753-3322(05)80044-7 16507391

[B4] Geoerger B Gaspar N Opolon P Morizet J Devanz P Lecluse Y Valent A Lacroix L Grill J Vassal G EGFR tyrosine kinase inhibition radiosensitizes and induces apoptosis in malignant glioma and childhood ependymoma xenografts Int J Cancer 2008 123 209 216 10.1002/ijc.23488 18386816

[B5] Michel Z Abderrahim Z David A Mahmut O The epidermal growth factor receptor (EGFR) in head and neck cancer: its role and treatment implications Radiation Oncology 2006 1 11 10.1186/1748-717X-1-11 16722544 PMC1524965

[B6] Wachsberger PR Burd R Cardi C Thakur M Daskalakis C Holash J Yancopoulos GD Dicker AP VEGF trap in combination with radiotherapy improves tumor control in U87 glioblastoma Int J Radiat Oncol Biol Phys 2007 67 1526 1537 17234361 10.1016/j.ijrobp.2006.11.011

[B7] Graham W Kris G Yong X Mahesh K William ST Clair Selectively enhanced radiation sensitivity in prostate cancer cells associated with proteasome inhibition Oncology Reports 2006 15 1287 1291 16596199

[B8] Lee YY Kao CL Tsai PH Tsai TH Chiou SH Wu WF Caffeic acid phenethyl ester preferentially enhanced radiosensitizing and increased oxidative stress in medulloblastoma cell line Childs Nerv Syst 2008 24 987 994 10.1007/s00381-008-0636-2 18470517

[B9] Helen PSW Le Y Emily KYL Emily KKT William KW Cho CH Nicotine Promotes Colon Tumor Growth and Angiogenesis through β-Adrenergic Activation Toxicological Sciences 2007 97 279 287 10.1093/toxsci/kfm060 17369603

[B10] Vermes I Clemens H Helga SN Chris R A novel assay for apoptosis Flow cytometric detection of phosphatidylserine expression on early apoptotic cells using fluorescein labelled Annexin V Journal of Immunological Methods 1995 17 39 51 10.1016/0022-1759(95)00072-I 7622868

[B11] Ahmed KM Li JJ ATM-NF-kappaB connection as a target for tumor radiosensitization Curr Cancer Drug Targets 2007 7 335 342 10.2174/156800907780809769 17979628 PMC4156318

[B12] Tamatani T Azuma M Motegi K Takamaru N Kawashima Y Bando T Cepharanthin-enhanced radiosensitivity through the inhibition of radiation-induced nuclear factor-kappaB activity in human oral squamous cell carcinoma cells Int J Oncol 2007 31 761 768 17786306

[B13] Voorhees PM Dees EC O'Neil B Orlowski RZ The proteasome as a target for cancer therapy Clin Cancer Res 2003 9 6316 6325 14695130

[B14] Adams J The proteasome: structure, function, and role in the cell Cancer Treat Rev 2003 29 3 9 10.1016/S0305-7372(03)00081-1 12738238

[B15] Xiong HQ Abbruzzese JL Lin E Wang L Zheng L Xie K NF-κB activity blockade impairs the angiogenic potential of human pancreatic cancer cells Int J Cancer 2004 108 181 188 10.1002/ijc.11562 14639600

[B16] Liao Z Komaki R Mason KA Milas L Role of cyclooxygenase 2 inhibitors in combination with radiation therapy in lung cancer Clin Lung Cancer 2003 4 356 365 10.3816/CLC.2003.n.015 14599301

[B17] Terakado N Shintani S Yano J Chunnan L Mihara M Nakashiro K Hamakawa H Overexpression of cyclooxygenase 2 is associated with radioresistance in oral squamous cell carcinoma Oral Oncol 2004 40 383 389 10.1016/j.oraloncology.2003.09.005 14969817

[B18] Arteaga C Targeting HER1/EGFR: a molecular approach to cancer therapy Semin Oncol 2003 30 3 14 12840796

[B19] Wu RR Wu SX Zhao C Xie FY Gao JM Hu WH Gao YH Li FY Cui TT Lu TX Phase II clinical trial of h-R3 combined radiotherapy for locoregionally advanced nasopharyngeal carcinoma Chin J Cancer 2007 26 874 879 17697551

[B20] Huang XD Yi JL Gao L Xu GZ Jin J Yang WZ Lu TX Wu SX Wu RR Hu WH Xie WC Han F Gao YH Gao JM Pan JJ Chen CB Lang JY Li T Dong Y Fu YB Fan L Li BS Li J Wang XH Chen BX Gao XS Zhang P Wu XW Hu BQ Multi-center phase II clinical trial of humanized anti-epidermal factor receptor monoclonal antibody h-R3 combined with radiotherapy for locoregionally advanced nasopharyngeal carcinoma Chin J Onco 2007 29 197 202 17649636

[B21] Bonner JA Harari PM Giralt J Azarnia N Shin DM Cohen RB Jones CU Sur R Raben D Jassem J Ove R Kies MS Baselga J Youssoufian H Amellal N Rowinsky EK Ang K Radiotherapy plus cetuximab for squamous-cell carcinoma of the head and neck Engl J Med 2006 354 567 578 10.1056/NEJMoa053422 16467544

[B22] Ferrara N Vascular endothelial growth factor as a target for anticancer therapy Oncologist 2004 9 2 10 10.1634/theoncologist.9-suppl_1-2 15178810

[B23] Hicklin DJ Ellis LM Role of the vascular endothelial growth factor pathway in tumor growth and angiogenesis J Clin Oncol 2005 23 1011 1027 10.1200/JCO.2005.06.081 15585754

[B24] Teicher BA Dupuis N Kusomoto T Robinson FM Liu F Menon K Coleman CN Antiangiogenic agents can increase tumor oxygenation and response to radiation therapy Rad Oncol Invest 1995 2 269 276 10.1002/roi.2970020604

[B25] Sclabas GM Uwagawa T Schmidt C Hess KR Evans DB Abbruzzese JL Chiao PJ Nuclear factor κB activation is a potential target for preventing pancreatic carcinoma by aspirin Cancer 2005 103 2485 2490 10.1002/cncr.21075 15861417

[B26] Takada Y Kobayashi Y Aggarwal BB Evodiamine abolishes constitutive and inducible NF-κB activation by inhibiting InBa kinase activation, thereby suppressing NF-κB-regulated antiapoptotic and metastatic gene expression, up-regulating apoptosis, and inhibiting invasion J Biol Chem 2005 280 17203 17212 10.1074/jbc.M500077200 15710601

[B27] Takada Y Murakami A Aggarwal BB Zerumbone abolishes NF-κB and InBa kinase activation leading to suppression of antiapoptotic and metastatic gene expression, upregulation of apoptosis, and downregulation of invasion Oncogene 2005 24 6957 6969 10.1038/sj.onc.1208845 16007145

